# The impact of the National HIV Health Care Worker Hotline on patient care in South Africa

**DOI:** 10.1186/1742-6405-8-4

**Published:** 2011-01-26

**Authors:** Briony S Chisholm, Karen Cohen, Marc Blockman, Hans-Friedemann Kinkel, Tamara J Kredo, Annoesjka M Swart

**Affiliations:** 1 Medicines Information Centre, University of Cape Town, Cape Town, South Africa; 2 Division of Clinical Pharmacology, University of Cape Town, Cape Town, South Africa; 3 HIV & TB Medicine Unit, Foundation for Professional Development, Pretoria, South Africa; 4 South African Cochrane Centre, MRC, Cape Town, South Africa

## Abstract

**Background:**

South Africa has a huge burden of illness due to HIV infection. Many health care workers managing HIV infected patients, particularly those in rural areas and primary care health facilities, have minimal access to information resources and to advice and support from experienced clinicians. The Medicines Information Centre, based in the Division of Clinical Pharmacology at the University of Cape Town, has been running the National HIV Health Care Worker (HCW) Hotline since 2008, providing free information for HIV treatment-related queries via telephone, fax and e-mail.

**Results:**

A questionnaire-based study showed that 224 (44%) of the 511 calls that were received by the hotline during the 2-month study period were patient-specific. Ninety-four completed questionnaires were included in the analysis. Of these, 72 (77%) were from doctors, 13 (14%) from pharmacists and 9 (10%) from nurses. 96% of the callers surveyed took an action based on the advice received from the National HIV HCW Hotline. The majority of actions concerned the start, dose adaption, change, or discontinuation of medicines. Less frequent actions taken were adherence and lifestyle counselling, further investigations, referring or admission of patients.

**Conclusions:**

The information provided by the National HIV HCW Hotline on patient-specific requests has a direct impact on the management of patients.

## Background

Medicine information services aim to promote the safe and effective use of medicines [1]. The Medicines Information Centre (MIC), which is based in the Division of Clinical Pharmacology at the University of Cape Town, has been in operation since 1980. Over the past 30 years the MIC has received a constantly increasing number of queries each year, with over 8 000 in 2008 and 2009.

With the public sector rollout of antiretroviral therapy (ART), in response to the HIV epidemic in South Africa, an increasing need for information on ART, especially for health care workers in rural areas with little access to expert advice, was identified. In 2008, an estimated 568 000 patients were enrolled for ART in South Africa - 449 000 in the public sector, 32 000 at Non-Governmental Organisations and 86 000 in the private sector (Disease Management and Workplace Treatment Programmes) [2]. The HIV and AIDS Strategic Plan for South Africa 2007-2011 recognises that no single sector, ministry, department or organisation can by itself be held responsible for the control of HIV and AIDS [3] and an integrated approach involving all sectors - public and private, health and non-health, is being promoted.

In March 2008 the MIC, in collaboration with the Foundation for Professional Development (FPD) and PEPFAR/USAID, established the National HIV Health Care Worker (HCW) Hotline. This toll-free hotline provides information to all health care workers in South Africa on aspects concerning the treatment of HIV infection and related diseases.

Queries are answered relating to a variety of topics including HIV testing, post exposure prophylaxis, the management of HIV in pregnancy, prevention of mother-to-child transmission, when to initiate therapy, treatment selection, recommendations for laboratory and clinical monitoring, how to interpret and respond to laboratory results, management of adverse events, drug interactions, treatment and prophylaxis of opportunistic infections, drug availability and adherence support. The hotline operates from Mondays to Fridays 8.30 am - 4.30 pm. The hotline number is 0800 212 506.

The queries are handled by four specially-trained drug information pharmacists who share more than 50 years of drug information experience between them. They have direct access to the latest information databases and reference sources, as well as to a network of experienced clinicians and consultants across South Africa, including specialists from the University of Cape Town's Faculty of Health Sciences, Groote Schuur Hospital and Red Cross War Memorial Children's Hospital. Queries requiring clinical input are discussed with experienced clinicians (just under 50% of queries received.)

Several previous studies have shown that both general drug information centres and HIV hot/warmlines can be a valuable source of information for health care workers [4,5]. One study from a developing country (Uganda) showed the successful establishment of an HIV warmline to a very small, rural area [6]. A telemedicine (e-mail) service for HIV/AIDS physicians, based in Belgium and supporting developing countries, whilst being perceived as valuable, has seen a decline in its use [7].

The objectives of this study were firstly to evaluate whether enquirers use the information given to them by the National HIV HCW Hotline. Secondly, we aimed to assess how the information provided impacted on patient care. Thirdly, we aimed to describe which categories of health care worker were making use of the service for patient-related queries. In addition, we wanted to identify differences in the type of question asked, and the action taken as a result of information given, between health care worker categories.

## Methods

The study was carried out between 3 August and 30 September 2009 at an independent drug information centre situated in the Division of Clinical Pharmacology at the University of Cape Town, on routine calls to the HIV Hotline.

The study received ethical approval from the University of Cape Town Faculty of Health Sciences Human Research Ethics Committee.

To evaluate the impact of the information on patient management, callers who asked patient-specific questions were contacted on the same day for a questionnaire-based interview on how they used the information.

For the duration of the study, the drug information pharmacists in the MIC gave the name and contact number of all HCWs who called the hotline with a patient-specific query, to a specially trained administrative assistant. The interview was conducted by her on the day the query was asked. Health care workers who did not have the time to answer the questions immediately were given the option of receiving a faxed or e-mailed questionnaire. Where health care workers called more than once in a day they were asked to complete the questionnaire only for the first patient-related query of that day.

The basic information collected in the questionnaire about the caller included the HCW category (doctors, nurses, pharmacists, others), the province from where they called and if they called from an urban or rural area. The callers were then asked what actions were taken out of a list of possible actions as a result of the information received, whether they found that the information provided was useful and whether they felt the availability of the hotline benefited their patient.

The respondents' willingness to complete the questionnaire was confirmed verbally and a positive response taken as indicative of their consent. No consequences accrued to respondents who decided not to participate. Confidentiality of responses was maintained throughout.

Data captured on the questionnaire were entered into an Excel^® ^spreadsheet, and analyses were performed using Excel^® ^and STATA version 10 (Stata corp. College Station, TX, USA).

## Results

There has been a steady increase in the number of calls to the hotline since its inception, with over 300 calls per month in May 2010.

During the two months of the study, 511 calls were received by the National HIV HCW Hotline, of which 224 (44%) were patient-specific queries. Table 1 shows the distribution of the calls received during the observation period among the different groups of HCWs.

**Table 1:** Distribution of calls among the different HCW groups

	Doctors	Nurses	Pharmacists	Other	TOTAL
**Patient specific questions**	167	31	26	0	224

**Other queries**	188	24	46	29	287

**TOTAL**	355	55	72	29	511

The main group of HCWs using the hotline were doctors with 355 (69%) calls. The number of calls/queries received from pharmacists, nurses and other HCWs was 72 (14%), 55 (11%) and 29 (6%) respectively.

Of the 511 total calls received over the period, the proportion of patient-specific to non-specific calls was highest amongst nurses, with 31/55 (56%) of their calls being patient-specific, followed by doctors 167/355 (47%) and pharmacists 26/72 (36%).

We managed to contact 187 of the 224 (83%) callers with patient-specific questions telephonically. Of these, 98/187 (52%) completed and returned questionnaires. Four of the 98 questionnaires were not included in the analysis. One questionnaire was completed in error, as the query was not patient-related. One questionnaire was incorrectly filled in because the doctor misunderstood the instructions and ticked all the actions, having used the service many times before, for several patients. She however commented that the service was very useful and ultimately benefitted her patients. The other two questionnaires that were not included in the analysis only contained information regarding the category of HCW and the area they worked in, the rest of the questionnaire was not filled in.

Of the remaining 94 questionnaires, 72 (77%) were received from doctors, 13 (14%) from pharmacists and 9 (9%) from nurses. There was no significant difference in response rate between HCW categories (Chi-squared p = 0.23.)

Fifty two (55%) of the 94 questionnaires were received from HCWs practicing in urban areas and 37 (40%) practicing in rural areas. In 5 (5%) questionnaires the area of origin was unknown.

Ninety of the 94 participants (96%) stated that an action was taken as a result of the information received. Some participants reported more than one action taken. Table 2 summarises the actions taken.

**Table 2:** Action taken as a result of the information received

Action taken	/94	%
**Medicine started**	45	48
ARVs	32	34
Co-trimoxazole	0	0
TB treatment	5	5
Other	6	6

**Medicine discontinued/interrupted**	54	57
ARVs	41	44
Co-trimoxazole	5	5
TB treatment	4	4
Other	3	3
**Reason for change**		
Adverse event	31	33
Failure	10	11
Interaction	5	5
Pregnancy	3	3
Other	3	3

**Dose changed**	18	19

**Schedule changed**	7	7

**Changed ARV Regimen**	32	34

**Admitted patient to hospital**	10	11

**Laboratory testing/further investigations**	33	35

**Referred patient to specialist clinic/service**	19	20

**Lifestyle changes**	17	18

**Adherence counselling**	36	38

Please note that some health care workers indicated that they had taken more than one action

The most common actions taken concerned the start (34%) and/or interruption or discontinuation (44%) of ART. The most common reasons for medicines to be interrupted or discontinued were adverse events (33%), treatment failure (11%) or drug interactions (5%). Dosing adjustments were done in 19% of patients.

Actions taken with regard to the management of the patients included further investigations such as laboratory testing (35%), referrals to specialist facilities (20%) and admission to hospitals (10%).

There was no significant difference between health care worker groups in terms of action taken.

Asked if the information provided by the National HIV HCW Hotline was useful or not, 92/94 (98%) found the information useful. One participant answered no, stating the information given was not timely enough and one did not fill in an answer. Fifty-seven participants (61%) found the information both useful for the management of the specific patient but also for teaching, research or personal knowledge. Twenty nine (31%) participants found the information useful for the management of the specific patient only and 5 (5%) participants found it useful because of other reasons (teaching, research, personal knowledge) only.

The last question on the questionnaire was: "Do you feel the availability of the hotline benefited your patient?" All participants except two, who did not complete this question, answered yes.

## Discussion

The National HIV HCW Hotline was established in March 2008, in response to the roll-out of ART in the public sector of South Africa, to provide information and decision support on the treatment of HIV infection and related diseases. Use of the service has consistently increased since its inception. We conducted the study over the specific 2 month period as it was 5 years since the rollout of ART. In addition, no major changes had been made to the guidelines.

The most frequent users of the HIV HCW Hotline are doctors, nurses and pharmacists, with doctors being the leading group, responsible for 355/511 queries (69%) during the study period. This may reflect the fact that medical doctors are still the predominant group involved in decisions around HIV treatment in South Africa.

About 44% of all callers over the study period had patient-specific questions. This highlights that the HIV HCW Hotline is frequently used as a resource to guide direct patient management decisions. Nurses had the highest proportion of patient-specific queries with 31/55 (56%), highlighting the important role the HIV HCW Hotline is playing in assisting nurses with regard to direct patient management.

Based on the results of the survey, almost all (96%) of the HCWs who called the National HIV HCW Hotline with a clinical query during August and September 2009 reported that they made a change to their management of their patient as a result of advice given.

Most of the actions that took place as a consequence of the advice given by the hotline concerned direct treatment related decisions such as initiation (34%), dose adjustments (19%), discontinuation (44%) or change of ART. However, a substantial proportion of actions also concerned the overall management of patients, such as the initiation of further diagnostic procedures, referrals to specialist services (20%) or hospital admissions (10%). This demonstrates that the HIV HCW Hotline is utilised not only for medicine specific questions but for the overall management of the patients.

Almost all callers interviewed confirmed that the information provided was useful and timely and to the benefit of the patient.

It is a limitation of this study that the response rate of the survey was only 52%. We cannot exclude a bias towards satisfied customers as unsatisfied customers might have been less willing to respond.

Based on the findings of this study, we conclude that the HIV HCW Hotline serves its purpose as a measure to strengthen the public health system through information, advice and decision support in the management of patients with HIV infection.

The current developments in the public health sector are characterised by efforts to drastically increase the number of HIV infected individuals on ART. Nurse initiated management of ART (NIMART) is a goal of the South African Department of Health [8], and nurses will increasingly be drawn upon to initiate and manage patients on ART.

Nurses form the bulk of HCWs at primary care level in under-resourced settings in sub-Saharan Africa [9]. They are frequently working in facilities where there is limited contact with doctors or pharmacists, and may be required to make prescribing and clinical management decisions with little support. They will be required to manage very ill patients, and will have to do so with far less clinical training than doctors. A telephonic helpline providing clinical advice and support may be a valuable resource to support nurses in fulfilling this clinical function.

## Conclusions

The National HIV HCW Hotline provides useful and timely information, advice and decision support that directly impacts patient management. It is an essential service to strengthen the public health system, especially for HCWs who work in remote areas with poor access to experts. The hotline will play an increasingly important role in the ambitious expansion of ART services in South Africa through NIMART.

## Competing interests

The authors declare that they have no competing interests.

## Authors' contributions

AS conceptualised the study, participated in the design and coordination and helped to draft the manuscript. BC participated in design and drafted the manuscript. KC and MB made a contribution to conception and design. KC performed statistical analysis. All authors revised the script critically and read and approved the final manuscript.
